# International Multispecialty Consensus on How to Evaluate Ultrasound Competence: A Delphi Consensus Survey

**DOI:** 10.1371/journal.pone.0057687

**Published:** 2013-02-28

**Authors:** Martin G. Tolsgaard, Tobias Todsen, Jette L. Sorensen, Charlotte Ringsted, Torben Lorentzen, Bent Ottesen, Ann Tabor

**Affiliations:** 1 Department of Obstetrics, Copenhagen University Hospital Rigshospitalet, Copenhagen, Denmark; 2 Centre for Clinical Education, Copenhagen University Hospital Rigshospitalet, Copenhagen, Denmark; 3 Department of Anesthesia, University of Toronto and The Wilson Centre, University Health Network and UoT, Toronto, Canada; 4 Department of Radiology, Herlev University Hospital, Herlev, Denmark; 5 Juliane Marie Centre, Copenhagen University Hospital Rigshospitalet, Copenhagen, Denmark; 6 Department of Obstetrics, Juliane Marie Centre, Copenhagen University Hospital Rigshospitalet, Copenhagen, Denmark; University of South Australia, Australia

## Abstract

**Objectives:**

To achieve international consensus across multiple specialties on a generic ultrasound rating scale using a Delphi technique.

**Methods:**

Ultrasound experts from Obstetrics-Gynaecology, Surgery, Urology, Radiology, Rheumatology, Emergency Medicine, and Gastro-Enterology representing North America, Australia, and Europe were identified. A multi-round survey was conducted to obtain consensus between these experts. Of 60 invited experts, 44 experts agreed to participate in the first Delphi round, 41 remained in the second round, and 37 completed the third Delphi round. Seven key elements of the ultrasound examination were identified from existing literature and recommendations from international ultrasound societies. All experts rated the importance of these seven elements on a five-point Likert scale in the first round and suggested potential new elements for the assessment of ultrasound skills. In the second round, the experts re-rated all elements and a third round was conducted to allow final comments. Agreement on which elements to include in the final rating scale was pre-defined as more than 80% of the experts rating an element four or five, on importance to the ultrasound examination.

**Results:**

Two additional elements were suggested by more than 10% of the experts in the first Delphi round. Consensus was obtained to include these two new elements along with five of the original elements in the final assessment instrument: 1) Indication for the examination 2) Applied knowledge of ultrasound equipment 3) Image optimization 4) Systematic examination 5) Interpretation of images 6) Documentation of examination and 7) Medical decision making.

**Conclusion:**

International multispecialty consensus was achieved on the content of a generic ultrasound rating scale. This is the first step to ensure valid assessment of clinicians in different medical specialties using ultrasound.

## Introduction

The usage of ultrasonography has expanded rapidly in many medical specialties over the last decades as smaller and less expensive ultrasound equipment has become available. Although ultrasound imaging traditionally is considered safe, its use is highly operator dependent. [1] The lack of sufficient operator skills can lead to diagnostic errors that eventually compromise patient safety due to unnecessary tests or interventions. [2] Consequently, there is a need to ensure competence of clinicians using ultrasound by assessing adequacy of their skills. [3], [4] Therefore, reliable and valid assessment instruments are needed to certify clinicians as well as to re-certify individuals, whose skills may have declined over time. [3] Different specialties may, however, present contrasting perspectives on what should be included in the assessment of ultrasound skills. The aim of this study was to explore whether it is possible to obtain international consensus across experts from multiple medical specialties on a generic rating scale for assessment of ultrasound competence. Such a rating scale would enable clinicians from different specialties to evaluate generalisable aspects of performance and provide a common foundation for assessment of ultrasound skills.

## Methods

### Study Design

A Delphi technique to obtain expert consensus on the content of a scale for assessment of ultrasound skills was used for this study. The Delphi technique is an anonymous structured approach, in which information is gathered from a group of participants (e.g. ultrasound experts) through a number of Delphi rounds. In the first round, participants evaluate and comment a number of elements of interest. Based on the group response, participants then re-evaluate these elements in subsequent Delphi rounds. This process is repeated until consensus has been reached. The web-based, anonymous nature of the Delphi technique ensures that a single individual cannot dominate the consensus formation and all participants are equally able to change their opinion in the course of the process [5]–[9].

The study originated from the Juliane Marie Centre, Copenhagen University Hospital Rigshospitalet, Denmark from February to May 2012. Written consent was obtained from all participants by e-mail and ethical approval from the regional ethical committee of the Capital Region (Protocol-number H-2-2012-038), Copenhagen, Denmark, was obtained before conducting the study.

### Selection of Experts and Specialties

Experts were identified according to selection criteria described by Palter et al. [8], [9] Criteria for inclusion in our study were that the experts: 1) were regarded as leaders in their field of practice, 2) actively practiced ultrasound on a regular basis and were involved in post-graduate training, 3) had strong publication records in ultrasound imaging, and finally 4) represented a broad geographical area including North America, Europe, and Australia.

There is no consensus on the number of experts required for a Delphi study, [8] although previous studies have used 5–10 participants from each professional group. [10] Consequently, a total of 60 experts from the following six specialties were invited: Radiology, Obstetrics-Gynaecology, Emergency Medicine, Rheumatology, Gastro-Enterology, and Surgery including Urology. A group of six leading members of the international ultrasound societies representing different specialties helped identify these 60 experts based on the criteria mentioned above.

### Drafting the Elements for the First Delphi Round

Key elements of the ultrasound examination were identified prior to the first Delphi round by reviewing existing research on imaging perception and assessment theory along with recommendations provided by the European and American ultrasound societies. [11]-[19] These sources of information were triangulated into a new framework containing seven key elements that formed the starting point of the first Delphi round. All elements were provided with short explanations and examples.

### The First Delphi Round

The experts agreed to participate by completing the first Delphi round consisting of an anonymised questionnaire. In this questionnaire, the experts were instructed to rate and, if relevant, comment the seven key elements on how important they consider them to be for assessment of trainees’ ultrasound skills. Each element was rated on a five-point Likert scale that was provided with response anchors (1 = Not relevant; 3 = Relevant but not essential; 5 = Essential). Finally, experts were encouraged to suggest up to three new elements that should be considered for assessment of ultrasound skills. All experts were contacted by e-mail. In each of the three rounds they had four weeks to respond during which two e-mail reminders at two-week intervals were sent to non-responders.

### The Second Delphi Round

All ratings from the first Delphi round were analysed and distributions of scores were presented in percent for each element. Any comments on elements from the first round were analysed and the descriptions of elements were re-phrased in case of ambiguity. Thus, the content of the elements remained unchanged but clarification of the wordings was allowed. All proposed new elements from the first round were categorized by two of the authors (MT & AT). New elements were coded and classified into groups describing similar subject matter. [20] Groups of elements that were proposed by more than 10% of the expert panel were included as new elements in the second Delphi round.

In the second Delphi round, the experts were informed about the distribution of scores and selected comments from the other members of the panel produced in the first Delphi round. They were instructed to re-consider the elements presented in the first round as well as to rate and comment the new elements the same way as in the first round.

The final content of the assessment instrument was based on consensus obtained after the second Delphi round. In previous Delphi studies, consensus was defined as more than 80% of the experts supporting an element. [7], [21], [22] Hence, an element was included when more than 80% of the experts regarded it as essential, which corresponded to an element being rated four or five out of five on importance for assessment of ultrasound skills. No elements were excluded between the first to the second round. This was done to allow experts to revise their opinion on elements from the first round when considering the ratings and comments provided by the other members of the expert panel.

### The Third Delphi Round

The elements included in the assessment instrument were given response anchors on five-point Likert scales. In the third round, participants were also provided the opportunity to comment the final outline of the assessment instrument including the response anchors. These comments were used to avoid ambiguity of response anchors and to ensure that the scores were aligned to similar performance characteristics through the rating scale. Even in case of no comments, all experts were asked to reply to monitor response rates.

### Statistical Analysis

All data were handled in SPSS ver. 19.0. Frequency of scores was calculated for each Delphi round. Kruskal-Wallis test was used to compare groups for differences between specialties and nationalities in the two first Delphi rounds and a post-hoc analysis using Bonferroni corrections was performed. Wilcoxon signed ranks test was used to compare ratings of the elements between the first and second Delphi round. Missing data points were excluded from comparative analysis listwise.

## Results

### Participants and Drafting the Initial Elements

**Figure 1:**
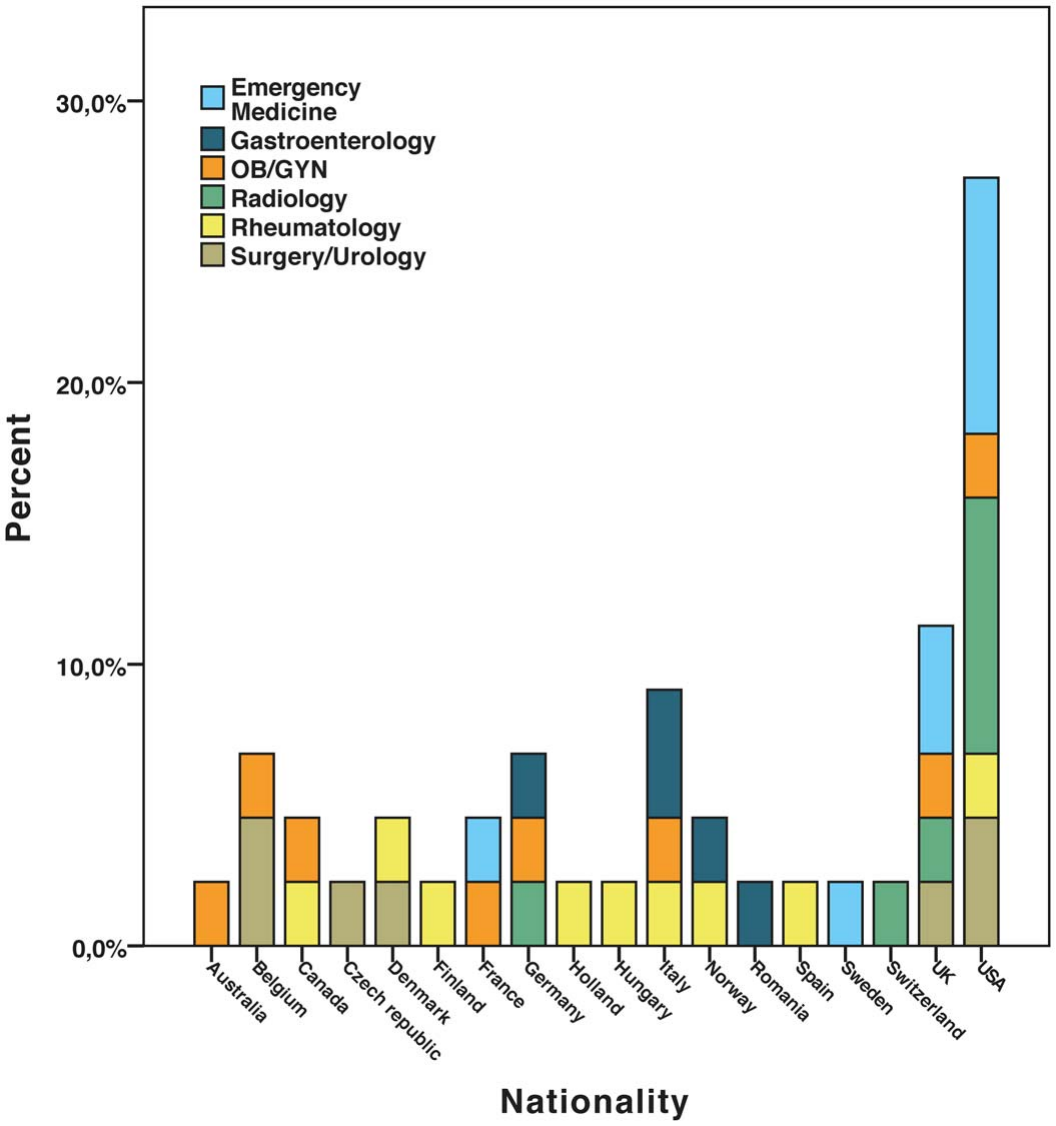
Baseline characteristics of experts agreeing to participate in the Delphi study.

Forty-four of the 60 experts invited agreed to participate in the first Delphi round (73.3%). In the second round, 41 of the 44 initial experts replied (93.2%) and of these, 37 responded in the third and final round (90.2%). The three non-responders in the second round were from Obstetrics-Gynaecology, Surgery/Urology, and Emergency Medicine. There was one non-responder in the third round from each of the following specialties: Obstetrics-Gynaecology, Emergency Medicine, Radiology, and Rheumatology. Baseline information on nationality and specialty of the experts is shown in Figure 1.

### The Delphi Rounds

The three Delphi rounds resulted in the *Objective Structured Assessment of Ultrasound Skills* (OSAUS) scale. Distribution of scores and selected comments from the experts in the first and second Delphi round are shown in Table 1. There were no missing values in the first Delphi round and 8.4% (31/369) missing values in the second Delphi round. New elements were suggested by 26 of the 44 participating experts in the first Delphi round. The mean number of new elements suggested was 1.2 (SD 1.2). Only two new elements were suggested by more than 10% of the 44 experts and hence included for further rating and commenting in the second Delphi round: *‘Indication for the examination’* (11.4%) and *‘Medical decision making’* (11.4%). Frequencies of the suggested new elements are shown in Table 2. Both of these two new elements along with five of the seven original elements were rated four or five by more than 80% of the experts in the second Delphi round and consequently included in the final assessment instrument. In the third Delphi round, the two new elements were revised to include the term *“If applicable”* to fit the different contexts of use. Response anchors were also modified based on comments in the final outline of OSAUS (Table 3).

**Table 1:** Distribution of scores and examples of comments from experts (n = 44) after the first Delphi round.

		Not Relevant	Relevant but not essential			Essential
	Score	1	2	3	4	5
*Applied knowledge of ultrasound equipment	Round 1	0.0%	2.3%	4.5%	11.4%	81.8%
	Round 2	0.0%	0.0%	8.1%	16.2%	75.7%
	Comments	“This is an essential first step”. “This is a fundamental component for all people scanning – beginners and experts”.				
*Image optimization	Round 1	0.0%	0.0%	4.5%	27.3%	68.2%
	Round 2	0.0%	0.0%	0.0%	33.3%	66.7%
	Comments	”This is an essential second step”. “Suboptimal images are a source of loss of information”.				
Time of examination and hand motions	Round 1	0.0%	7.3%	43.9%	39.0%	9.8%
	Round 2	0.0%	8.1%	48.6%	40.5%	2.7%
	Comments	”Duration of the examination is not essential because even for an expert difficult cases may need long examination time”. “Nice to have as a skill, but not really that important”.				
*Systematic examination	Round 1	0.0%	2.3%	6.8%	31.8%	59.1%
	Round 2	0.0%	2.7%	2.7%	37.8%	56.8%
	Comments	”A systematic approach is important, particularly when training”. “A systematic approach should be pursued whenever possible”.				
Establishing patient cooperation	Round 1	2.3%	2.3%	22.7%	47.7%	25.0%
	Round 2	0.0%	5.6%	38.9%	50.0%	5.6%
	Comments	”To inform patients about steps of the examination can be important but not essential”.” This will develop with time - may not be an essential part of the examination”.				
*Interpretation of images	Round 1	0.0%	0.0%	0.0%	0.0%	100%
	Round 2	0.0%	0.0%	0.0%	2.8%	97.2%
	Comments	”This is what it is all about”.” If findings cannot be interpreted correctly, the exam loses its value, so this is essential”.				
*Documentation of examination	Round 1	0.0%	4.5%	6.8%	27.3%	61.4%
	Round 2	0.0%	0.0%	5.4%	24.3%	70.3%
	Comments	”The only way to transmit information (positive or negative) is to write a report!”. “I think there absolutely needs to be written communication”.				
New elements						
*Indication for the examination	Round 2	0.0%	0.0%	17.1%	24.4%	58.5%
	Comments	“The provider of the US may not be the interpreter/clinical decision maker (i.e. if a trainee is involved), but certainly the team should have a reason why the test is being performed”.				
*Medical decision making	Round 2	0.0%	2.4%	12.2%	24.4%	61.0%
	Comments	“It depends on the aim of the scan”. “Depends on who is scanning but this could be a negative thing if the 'sonographer' (doctor or not) gives advice and is not the treating healthcare practitioner.”				

Elements that were included in the final assessment instrument are marked with an asterisk*.

**Table 2:** New elements suggested by experts in the first Delphi round.

New element	Frequency, *n*
Medical history/indication for the examination	5*
Medical decision making	5*
Self-evaluation and knowledge of limitations of skills	3
‘As Low As Reasonably Achievable’ principle	2
Patient information and communication	2
Interventional skills	2
Other (e.g. area-specific elements, number of scans, use of quality criteria for images)	16

* Included in the second Delphi round.

**Table 3:** The Objective Structured Assessment of Ultrasound Skills (OSAUS) in its final form.

**1. Indication for the examination**	1	2	3	4	5
If applicable. Reviewing patient history and knowing why the examination is indicated.	Displays poor knowledge of the indication for the examination		Displays some knowledge of the indication for the examination		Displays ample knowledge of the indication for the examination
**2. Applied knowledge of ultrasound equipment**	1	2	3	4	5
Familiarity with the equipment and its functions, i.e. selecting probe, using buttons and application of gel.	Unable to operate equipment		Operates the equipment with some experience		Familiar with operating the equipment
**3. Image optimization**	1	2	3	4	5
Consistently ensuring optimal image quality by adjusting gain, depth, focus, frequency etc.	Fails to optimize images		Competent image optimization but not done consistently		Consistent optimization of images
**4. Systematic examination**	1	2	3	4	5
Consistently displaying systematic approach to the examination and presentation of relevant structures according to guidelines.	Unsystematic approach		Displays some systematic approach		Consistently displays systematic approach
**5. Interpretation of images**	1	2	3	4	5
Recognition of image pattern and interpretation of findings.	Unable to interpret any findings		Does not consistently interpret findings correctly		Consistently interprets findings correctly
**6. Documentation of examination**	1	2	3	4	5
Image recording and focused verbal/written documentation.	Does not document any images		Documents most relevant images		Consistently documents relevant images
**7. Medical decision making**	1	2	3	4	5
If applicable. Ability to integrate scan results into the care of the patient and medical decision making.	Unable to integrate findings into medical decision making		Able to integrate findings into a clinical context		Consistent integration of findings into medical decision making

Differences in scores between nationalities, specialties, and scores in the first two Delphi rounds were examined as shown in Table 4. A statistically significant difference between specialties was found regarding one element – ‘Documentation of examination’ (p = 0.034). The median score of this element was four or five in all specialties and 94.6% of all experts scored this element four or five. Thus, this difference had no relevance to the decision of inclusion of the element or not. The post-hoc analysis using Bonferroni corrections did not show any statistically significant differences between specialties. No statistically significant differences were detected between countries. Statistically significant differences between ratings in the first and second Delphi round were found regarding two elements – ‘Establishing patient cooperation’ (p = 0.049) and ‘Documentation of examination’ (p = 0.038).

**Table 4:** Differences in scores across specialties, nationalities, and between the first two Delphi rounds.

Original elements	Specialties (p-values)		Nationalities (p-values)		Between rounds (p-values)
	Round 1	Round 2	Round 1	Round 2	Round 1∶2
Applied knowledge of ultrasound equipment	0.45	0.97	0.51	0.85	0.80
Image Optimization	0.31	0.86	0.52	0.77	0.76
Time of examination and hand motions	0.14	0.73	0.56	0.11	0.46
Systematic examination	0.43	0.92	0.29	0.12	0.66
Establishing patient cooperation	0.31	0.07	0.71	0.57	0.049*
Interpretation of images	1.00	0.53	1.00	0.19	0.32
Documentation of examination	0.45	0.034*	0.39	0.18	0.038*
**New elements**					
Indication for the examination	Not included inRound 1.	0.47	Not included inRound 1.	0.22	Not included inRound 1.
Medical decision making	Not included inRound 1.	0.18	Not included inRound 1.	0.62	Not included inRound 1.

Statistically significant differences are marked with an asterisk*.

## Discussion

International multispecialty consensus on how to evaluate ultrasound skills was achieved using a Delphi technique. The resulting scale – the *Objective Structured Assessment of Ultrasound Skills* (OSAUS) – comprises seven elements describing essential sub-steps of an ultrasound examination. Consequently, the OSAUS scale possesses content validity [23] in terms of expert consensus on content of the scale and accordance with recommendations from the European and American ultrasound societies [18], [19].

This study suggests that one generic assessment instrument can be used to evaluate ultrasound skills in multiple clinical settings and disciplines. Although disagreement was anticipated between radiologists and clinicians as well as between medical and surgical specialties, no relevant differences were observed between these groups. Several assessment instruments designed for specific ultrasound procedures and examinations have previously been described. [22], [24], [25] For clinical use, however, it may not be feasible to develop a number of detailed assessment instruments for every conceivable medical setting because both trainee and assessor need to be familiar with the use of the instrument. Further, the use of elaborate and procedure-specific checklists may not always provide a better estimate of performance than scales that rely on general competencies. [26]–[28] In the assessment of ultrasound performance, the generic skills needed for competence are the same across different specialties according to recommendations from the ultrasound societies. [18], [19] Hence, one single rating scale for assessing ultrasound skills – such as the OSAUS – may be used in multiple specialties without compromising the ability to discriminate between levels of competence.

Acknowledging that the number of ultrasound scans needed for proficiency varies greatly, [29]–[31] the OSAUS scale provides a common ground for competency-based assessment. Previous research on clinician training requirements has been concerned with the number of cases needed for competence before starting working on-call or independent practice. [32], [33] Hence, the European and American ultrasound societies have recommended around 300 supervised examinations before independent practice. [18], [19] Depending on the type of examination, this may result in insufficient competency levels for some trainees, while it may exceed the training requirements for other trainees due to differences in learning curves. [30], [31] This underlines the need for instruments that enable assessment of trainee performance over time until proficiency rather than relying on a predetermined number of procedures. Consequently, valid assessment of ultrasound skills is essential to improve patient safety by reducing potential diagnostic errors made by clinicians, who are not sufficiently trained before using ultrasound. [2] Moreover, using a generic rating scale such as OSAUS for in-training assessment may also improve skills acquisition of clinicians during training due to structured formative feedback.

The Delphi technique is considered an excellent method to obtain consensus as well as to produce new ideas. However, it has also been criticized because the investigators to some extent control the content and number of questions in the survey. [8], [34] Previous studies have tried to compensate for this by producing a very elaborate list of sub-steps for subsequent evaluation by the expert panel, which lead to lower response rates and inadequate sampling. [8] To accommodate for these potential limitations we encouraged all experts in the first round to suggest new elements of relevance to the assessment of ultrasound skills. A cut-off set at 10% was used to include only those elements that were proposed by several ultrasound experts. Two non-technical skills – *‘Indication for the examination’* and *‘Medical decision making’* – were suggested by more than 10% and were eventually included in the final scale. Due to comments in the second iteration of the study, these elements were made optional to enable the scale to fit situations, in which the sonographer is not the clinician responsible for the care of the patient. This was, however, a subject that caused disagreement between experts judged by the comments provided in the second iteration of the study, although more than 80% agreed to include the elements. By including experts from multiple specialties and from Europe, North America, and Australia, some disagreement regarding content of a rating scale was expected. Significant differences in scores were only detected on one element in the second round of rating but this had no consequence for the decision to include the element or not. However, statistically significant changes in scores from the first to the second Delphi round were found on two elements (‘Establishing patient cooperation’ and ‘Documentation of examination’), indicating that the experts did in fact re-evaluate elements based on the comments and ratings provided in the second Delphi round.

This study has some potential limitations in terms of selecting experts and including specialties. Regarding sampling of experts, this study included more than twice the number of experts reported in similar Delphi studies to ensure adequate representation across specialties. [8], [9], [22] Further, the number of specialties included in this study was large but did not comprise an exhaustive list of all specialties using ultrasound. However, the aim of this study was not to examine ultrasound training requirements in all specialties rather than to draw upon the opinions of leading experts, who represent different approaches to ultrasound training. Studies are needed to evaluate how this novel rating scale can be used in different clinical specialties including how well the scale discriminates between different levels of competence. Adding an overall global rating score may allow different clinicians to perform a ‘tacit weighting’ of the elements and thereby a more precise estimate of performance [28].

## Conclusion

International multispecialty consensus was achieved on the content of a generic ultrasound rating scale. The resulting rating scale – the Objective Structured Assessment of Ultrasound Skills – is based on consensus between radiologists, physicians, and surgeons representing the various uses of ultrasound. This is the first step to ensure valid in-training assessment in multiple different medical specialties using ultrasound and thereby to ensure competency-based ultrasound training.
